# Supplementary material for: Bayesian model averaging for nonparametric discontinuity design
Source: PLoS One. 2022 Jun 30;17(6):e0270310. doi: 10.1371/journal.pone.0270310 (PMC9246148; doi:10.1371/journal.pone.0270310)
Supplement: Attachment — Submitted filename: Response to Reviewers.pdf [file pone.0270310.s001.pdf]

Response to Reviewers

We would like to thank the reviewers for their positive feedback and helpful comments on our manuscript. Below, we discuss the comments raised by the reviewers one by one. Throughout our revised manuscript, changes are indicated in orange text. Quotations from the editor and reviewers are indicated in blue text.

Our comments and adjustments based on the journal requirements as pointed out by the editor are listed after the response to the reviewers.

Reviewer #1

1. "In the abstract section, the quantification proposed by the authors supporting the innovation point of either model is crucial to solve the main problem of the average of Bayesian models, because the prior probabilities of different models greatly affect the results of the Bayesian model averaging method, and the authors please describe them in detail in this section."

   The reviewer raises a valid point. We have updated the following text to the description of the BNDD framework to stress that where we have used a uniform prior over the models, alternative priors may be chosen, for instance to address the problem of multiple comparisons when multiple RD analyses are run in parallel:

   "This approach ignores the uncertainty in the model posterior $p(M \mid D) = \frac{p(D|M)p(M)}{\sum_i p(D|M_i)p(M_i)}$, where $p(M_i)$ is the prior probability of model i. Ignoring the uncertainty in this distribution results in an overconfident overestimate of the effect size, and consequently of too optimistic conclusions of the efficacy of an intervention."

   and

   "Note that for now, we assume a uniform prior over the models, such that $p(M_0) = p(M_1) = 1/2$, but this may be changed, for instance to account for multiple comparisons (Guo & Heitjan, 2010)."

   We then return to this statement in our discussion section, where we now write:

   "The Bayesian model averaging procedure that we use in BNDD depends on the model probabilities $p(M_0)$ and $p(M_1)$. Here, we have assumed a uniform prior on these model probabilities, as we have no reason to prefer either the continuous null model or the discontinuous alternative. However, it should be noted that prior beliefs may be incorporated to reflect our initial assumptions on the probability of an effect, as well as to adjust for multiplicity in case many hypotheses are tested simultaneously (Guo & Heitjan, 2010) (for instance, in (Lansdell & Kording, 2019) a regression discontinuity design is used to test the causal influence between neuronal populations)."

2. "Is this article more applicable to the discontinuity model? The study of continuous models is not comprehensive and detailed, does this paper focus on the description of discontinuous models?"

Our method is intended to compare two regressions: one that contains a continuous latent function, and one that contains a discontinuous one. It is possible to use discontinuous covariance functions for either, such as a white noise (or constant) covariance function. In that case, BNDD is only able to detect a difference in the means pre- and post-intervention, which essentially recovers a z-test. This could be an interesting addition in cases where it is unclear whether there is any autocorrelation in the latent function.

3. "It is noteworthy that your paper requires careful editing of the format. There are many problems in the paper format, such as the first line of the paragraph, multiple syntax errors, etc."

   We apologize for the formatting errors in our manuscript. We have updated the manuscript throughout (please see the updates in orange). Specifically, we have
   - Removed the erroneous \todo{} comment in the first line.
   - Listed our department before our institute in the author affiliations section.
   - Added figure titles.
   - Put some equations in display mode rather than in text mode (new equations (12) and (13)).
   - Made small textual/grammatical corrections.

Reviewer 2

"One minor doubt for me is the paper assume the distribution is Gaussian distribution, and thus Gaussian process is used in the paper. Is possible real world simulation is not under Gaussian distribution, and thus can not be used in Gaussian process?"

The reviewer raises an interesting question that can be approached in two ways: in light of either the observation model, or the latent function. We address this with a new section in our discussion, which reads:

"Throughout this paper, we have assumed a Gaussian likelihood. This conveniently leads to an analytic solution of the GP posterior, because the GP prior is conjugate to this likelihood. However, using variational inference or the Laplace approximation, BNDD can be used in combination with non-Gaussian observation models (Rasmussen & Williams, 2005). For instance, one could use a Poisson likelihood to model observed count data (Adams et al., 2009), or a Bernoulli likelihood for binary observations (Williams & Barber, 1998). Furthermore, other nonparametric priors over the latent functions may be used, such as the Student t- process (Shah et al., 2014)."

Journal requirements

1. "Please ensure that your manuscript meets PLOS ONE's style requirements, including those for file naming."

   We have renamed our submission files and updated our manuscript to meet the PLOS ONE style requirements. Please let us know if there are any mistakes.

2. "We noticed you have some minor occurrence of overlapping text with the following previous publication(s), which needs to be addressed:

- https://arxiv.org/abs/1911.06722"

Note: we have contacted the editorial office regarding this matter (Carl Williams). The previous publication here concerns a **preprint** of the current submission, and has not previously been published. This preprint document is identical to the one we submit here (modulo the changes due to this revision).

3. "We note that Figure 5 in your submission contain map images which may be copyrighted."

Note that we have also contacted the editorial office regarding this issue as well. Fig. 5 is our own original contribution. It is based on the municipality and country borders data as provided by the Dutch Central Bureau of Statistics (CBS; 'Centraal Bureau voor Statistiek'), which are free to use if the CBS is credited. Furthermore, the election results are provided at the government website https://data.overheid.nl under the CC0 1.0 license. This has been made more explicit in the manuscript with the added section:

"First, the vote distribution per Dutch municipality were collected from the Dutch government website (Kennis- en exploitatiecetrum officiële overheidspublicaties, 2018). We then manually constructed an approximation of the phantom border (see the dashed lines in Fig. 5) and used this as a function to divide the available municipalities in either above or below the border. For visualization of country and municipality borders, data from the Dutch national georegister was used (CBS, 2022)."

in addition, we have incorporated this information in the figure caption, which now reads:

Phantom-border effects on populist voting. Discontinuity analysis along a two-dimensional boundary (indicated by the dashed line). **A**. Circles indicate the observed fraction of populist votes; municipalities are shaded according to the Gaussian process predictions. **B**. The distribution of effect size conditioned on M_1, $p(d \mid D, M_1)$, along the phantom border. The shaded interval indicates one standard deviation around the mean. The country and municipality border data are available at the website of the Dutch national georegister [46], and the superimposed populist voting fractions were derived from the 2017 election results at https://data.overheid.nl.
